# The Diet and Haemodialysis Dyad: Three Eras, Four Open Questions and Four Paradoxes. A Narrative Review, Towards a Personalized, Patient-Centered Approach

**DOI:** 10.3390/nu9040372

**Published:** 2017-04-10

**Authors:** Giorgina Barbara Piccoli, Maria Rita Moio, Antioco Fois, Andreea Sofronie, Lurlinys Gendrot, Gianfranca Cabiddu, Claudia D’Alessandro, Adamasco Cupisti

**Affiliations:** 1 Dipartimento di Scienze Cliniche e Biologiche, University of Torino, 10100 Torino, Italy; 2 Nephrologie, Centre Hospitalier le Mans, Avenue Roubillard, 72000 Le Mans, France; mariaritamoio@gmail.com (M.R.M.); andreea.sofronie@gmail.com (A.S.); lurlygendrot@gmail.com (L.G.); 3 Nefrologia, Ospedale Brotzu, 09100 Cagliari, Italy; antiocofois@gmail.com (A.F.); gianfranca.cabiddu@tin.it (G.C.); 4 Nefrologia, Università di Pisa, 56100 Pisa, Italy; dalessandroclaudia@gmail.com (C.D.); adamasco.cupisti@med.unipi.it (A.C.)

**Keywords:** diet, dialysis, haemodialysis, protein energy wasting, serum albumin Kt/V, dialysis efficiency, phosphate, survival

## Abstract

The history of dialysis and diet can be viewed as a series of battles waged against potential threats to patients’ lives. In the early years of dialysis, potassium was identified as “the killer”, and the lists patients were given of forbidden foods included most plant-derived nourishment. As soon as dialysis became more efficient and survival increased, hyperphosphatemia, was identified as the enemy, generating an even longer list of banned aliments. Conversely, the “third era” finds us combating protein-energy wasting. This review discusses four questions and four paradoxes, regarding the diet-dialysis dyad: are the “magic numbers” of nutritional requirements (calories: 30–35 kcal/kg; proteins > 1.2 g/kg) still valid? Are the guidelines based on the metabolic needs of patients on “conventional” thrice-weekly bicarbonate dialysis applicable to different dialysis schedules, including daily dialysis or haemodiafiltration? The quantity of phosphate and potassium contained in processed and preserved foods may be significantly different from those in untreated foods: what are we eating? Is malnutrition one condition or a combination of conditions? The paradoxes: obesity is associated with higher survival in dialysis, losing weight is associated with mortality, but high BMI is a contraindication for kidney transplantation; it is difficult to limit phosphate intake when a patient is on a high-protein diet, such as the ones usually prescribed on dialysis; low serum albumin is associated with low dialysis efficiency and reduced survival, but on haemodiafiltration, high efficiency is coupled with albumin losses; banning plant derived food may limit consumption of “vascular healthy” food in a vulnerable population. Tailored approaches and agreed practices are needed so that we can identify attainable goals and pursue them in our fragile haemodialysis populations.

## 1. Diet and Dialysis: A Changing Picture

The history of dialysis and the nutritional approach in dialysis are closely connected; over time, the definition of dietary needs and limitations has reflected the major advances in renal replacement therapy and the changes that have taken place in the demography of the dialysis population.

In this review we will attempt to contextualize some open question related to the diet and dialysis dyad. We will start by describing the three “eras” of diet-in-dialysis, each marked by a battle against a different threat to patients’ lives, and each associated with different problems, restrictions and indications (Table 1).

We will then discuss some questions still being debated and problems that have remained unsolved, trying to indicate the practical implications of some of the open issues and paradoxes (Table 2).

## 2. Dialysis and Diet: Different Enemies in Different Periods

### 2.1. The First Era: Potassium as the Killer

Hyperkalemia and salt and water overload can prove fatal to dialysis patients, and in the early days of dialysis, acute pulmonary oedema, severe hyperkalaemia and the co-occurrence of the two conditions were frequent, and often deadly [1,2,3]. Due to the limited efficiency of dialysis, some dietary habits often had fatal consequences. Acidosis, only later recognized as a main cause of increases in pre-dialysis potassium, often failed to be diagnosed [4,5]. Particularly until the mid-seventies, when dialysis became more widely available, dialysis start often followed a period of low-protein diet, generally one that was far more rigid and draconian (“diet or die”) than the ones now prescribed [6,7,8,9,10,11,12]. Growing awareness of the loss of advantage (and increase of side effects) of protein restriction after dialysis start set the stage for recommending a high-protein diet immediately after dialysis start.

Due to the high potassium content of much animal-derived food, which was preferred for its higher protein content, the patient’s intake of plant-derived food was reduced, often to the point of imposing a ban on fruit and vegetables [3,13,14]. In the same period, patients were often advised to follow low-sodium diets, to limit thirst and weight gain, and were given long lists of forbidden foods, including most plant-derived food products [13,15,16,17]. This resulted in life-saving but monotonous and complex diets [3,13].

Increased dialysis efficiency, together with an ageing dialysis population, with lower nutritional intake, has required that the problem be seen from a new perspective, and death due to hyperkalaemia has become less frequent; where hyperkalaemia remains as a problem, it is generally linked to acidosis, gastrointestinal bleeding, uncontrolled diabetes, or iatrogenic effects [18,19,20,21]. The present focus on dietary habits is more on avoidance of added potassium in processed foodsthan on limiting consumption of plant-derived aliments, whose importance for cardiovascular health is increasingly recognized [3,13,20,21,22,23,24,25,26].

### 2.2. The Second Era: Phosphate as the Silent Killer

Increased survival rates brought to light clinical problems evident only in the long term, summarized as rapid vascular aging in dialysis patients [27,28,29,30,31,32]. These conditions were sometimes called survivors’ diseases, to emphasize that they had emerged only when the problem of short-term mortality had been at least partially overcome. Imbalances in the calcium-phosphate-PTH axis were identified as the main cause of rapid vascular ageing, and a high calcium-phosphate product was, and still is, associated with vascular calcification, and increased risk of death [33,34,35,36,37,38,39].

As a consequence, dialysis patients received a new, even longer list of foods they had to cut down on, potentially leading to the paradox of (protein) malnutrition induced by focusing on avoidance of phosphate-rich food. Only recently has attention focused on added phosphorus, distinguishing between processed and unprocessed foods [13,14,40,41,42,43,44,45,46,47].

Hyperphosphatemia is not only the result of a nutritional pathogenesis, as it can be the result of several conditions in which dietary restrictions are of scarce benefit, while they are frustrating for patients and may even worsen protein-energy wasting. Hyperphosphatemia may be due to:

- a low dialysis dose, which is associated with higher mortality; conversely, hyperphosphatemia is usually corrected with long dialysis sessions, or daily dialysis [48,49];
- uncontrolled hyperparathyroidism is not responsive to dietary measures; furthermore, it is often connected with long dialysis vintage, which is in turn associated with poor nutritional status and poor outcomes [50,51,52,53];
- a low PTH level (usually post-parathyroidectomy), is often a sign of adynamic bone disease, another condition associated with impaired nutritional status and poor outcomes [54,55,56];

Conversely, in thrice-weekly dialysis, low pre-dialysis phosphate levels are often a sign of protein energy wasting, and are associated with poor survival, especially if combined with hypoalbuminemia [57,58]. The implementation of new treatments has to some extent diverted attention from diet, while a new player, malnutrition, has emerged on the scene.

### 2.3. The Third Era: Malnutrition as the Killer

The ageing of the dialysis population and the possibility of attaining long-term survival through dialysis and transplantation, has focused attention on the central role of malnutrition both in the incident population (elderly, high comorbidity), and as a characteristic of patients with a long treatment vintage [53,55,56,57,59,60,61,62,63]. Among the biomarkers associated with survival (Kt/V, Ca-P-PTH, acidosis, etc.), the strongest is serum albumin, where a low value represents the most robust metabolic risk marker [64,65,66,67,68,69,70,71,72,73].

In keeping with the importance of nutrition in dialysis, total cholesterol, another robust marker of nutrition, is the second-most reliable marker of survival, with a “U” shaped curve, as will be discussed in the case of obesity, the paradigm of reverse epidemiology [74,75]. Malnutrition is not merely the result of insufficient food intake, or of insufficient dialysis, but may reflect cardiovascular comorbidity, associated with inflammation (MIA, malnutrition, inflammation and artherosclerosis syndrome) [59,60,76,77,78]. As further discussed, the stronger the association with cardiovascular disease and inflammation, the harder it is to correct malnutrition: eating more may not be enough (Table 2 and Table 3).

## 3. Diet and Dialysis: Four Questions and Four Paradoxes

### 3.1. First Question: Are the “Magic Numbers” Still Valid?

A series of reference numbers were progressively integrated into the indications and guidelines for the nutritional management of dialysis patients [79,80,81,82,83,84,85,86]. Some of these may prove to be too high and others too low. In their updated and extensive recent review of the revised nutritional recommendations, Biurete and coworkers discuss the changing attitudes to renal diet (and to diet in the overall population), and Kalantar-Zadeh wonders if there is anything left to eat for our patients [13]. Some of the “magic numbers” set targets that are probably too high and those which are probably too low to be attained in present clinical practice [13,22]:

Targets that may be too high:

- Energy: 30–35 kcal/kg of dry body weight
- Proteins: at least 1.2 g/kg of dry body weight
- Phosphate: 800–1000 mg/day

Targets that may be too low, especially when diuresis is partly preserved:

- Water: as little as possible
- Sodium: not univocal; older guidelines recommend less than 100 mEq/day (5.8 g of NaCl)
- Potassuim: 2 g/day

It is interesting to note, however, that many of these often-cited numbers are either opinion based, or derived from old studies, often ones that involved a relatively small number of cases, whose characteristics no longer correspond to the profile of the patients now seen in our dialysis wards.

### 3.2. Second Question: Type of Dialysis Technique: Does It Matter?

Most nutritional indications on extracorporeal dialysis are based on the metabolic needs of “conventional” thrice-weekly bicarbonate-buffered haemodialysis [79,80,81,82,83,84,85,86]. However, there is increased acknowledgement that if dialysis is more frequent and/or sessions are longer, metabolic balance and nutritional status are more easily restored [87,88,89,90,91]. The option of increasing dialysis frequency should therefore be considered as a valid strategy for improving nutritional status.

A growing body of evidence on an extreme, albeit rare condition, pregnancy in dialysis patients, indirectly shows the importance of the duration and frequency of treatment in maintaining physiological balance and optimal nutritional status [92,93].

The diet-dialysis dyad is therefore modulated by dialysis prescriptions:

- On conventional thrice-weekly dialysis, dietary restrictions follow the need to maintain good metabolic balance (diet has to follow dialysis);
- On intensive dialysis schedules, higher efficiency and/or frequency may make an unrestricted diet possible (dialysis liberalizes diet);
- A particular case regards haemodiafiltration, in which high efficiency may be associated with significant protein loss, and the role of this loss in the context of dialysis-related malnutrition has still to be elucidated [94,95,96,97,98,99].

Overall, the current indications for the management of nutritional status in haemodialysis regard non-diabetic adults on thrice-weekly haemodialysis of “standard duration”. Elderly and diabetic patients were not included in the studies which defined our reference numbers; however, the definition of “old age” has varied over time, from being over 50 in the 1970s, to being over 80 (sometimes over 90) now. The same indications do not necessarily apply to children, incremental dialysis, quotidian or nightly dialysis (and all variations of frequency), haemodiafiltration or haemofiltration, while controversy exists on peritoneal dialysis, which is beyond the scope of this review [100,101].

### 3.3. Third Question: Processed or Unprocessed Food: What Are We Actually Eating?

The current approaches to nutritional counseling in dialysis are based on the calculation of the nutritional content of raw, unprocessed food. However, in the processed and preserved food products now widely consumed, the quantity of phosphate and potassium, and of other preservatives, taste enhancers and chemical colorants may be high, which means that a processed food product differs considerably from the natural one. In particular in the case of phosphate, it is not only the quantity that matters, but also the quality, as added inorganic phosphate is usually rapidly absorbable, and therefore more dangerous [102,103,104,105,106,107,108,109,110,111].

In this regard, the warnings on red meat are linked to those on processed meat, and the question is whether the risks are due to the additives and preservatives used or to the red meat itself [107,108,109,110,111].

The issue is even more complex if pesticides and trace elements are considered. For instance, fish fed with animal proteins are extremely rich in phosphate, in contrast with wild fish. However, the high content of heavy metals, mercury in particular, in large wild fish is also known, and another high risk category, that of pregnant women, are counseled to avoid eating them, as mercury can impede foetal growth. While our knowledge of such hidden dangers in the overall population is constantly accumulating, the little that is known about dialysis patients identifies them as a population at high risk for the chronic poisoning agents of our polluted world [112,113,114,115,116].

### 3.4. Fourth Question: How Should Malnutrition Be Seen?

When protein energy wasting was identified as one of the major causes of death in dialysis, the “under-dialysis syndrome” was often seen as the basis of this deadly condition [59,60,76,77,78,117,118]. The first studies on daily and nightly dialysis supported this interpretation [119,120,121,122,123,124,125,126]. The hypothesis advanced was therefore that eating better and more was possible and should be accompanied by better dialysis, and with attention to control of metabolic acidosis [117,118,127,128,129,130].

The aging of the dialysis population revealed the importance of new players: diffuse vascular disease and chronic inflammation merged with malnutrition in the malnutrition, inflammation and atherosclerosis (MIA) syndrome [59,60,76,77,78,131,132,133,134,135].

**Figure 1:**
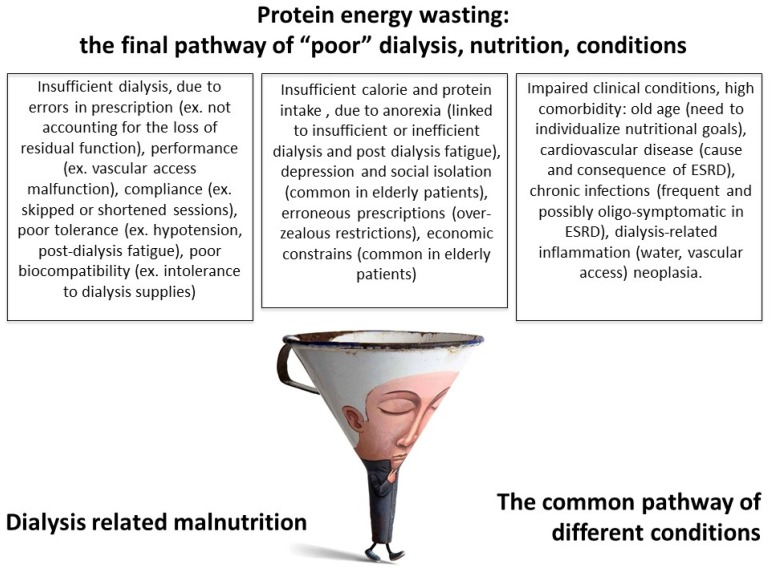
The two faces of malnutrition.

Hence, two different forms of malnutrition were identified:

- Protein energy wasting due to “poor nutrition”, related to low nutrient intake, in turn linked to incomplete correction of metabolic balance, insufficient dialysis, non biocompatible membranes, poorly controlled hypertension, combined with an overly restricted diet. This form of malnutrition generally responds to nutritional intervention, after optimization of the dialysis schedule.
- Protein energy wasting as a result of poor clinical conditions: the prototype is the MIA syndrome, mentioned above. This type of malnutrition is less responsive to nutritional interventions and its prognosis is linked to the patient’s life expectancy (Figure 1).

## 4. Diet and Dialysis: Four Paradoxes

### 4.1. First Paradox: Energy Intake and Obesity

**Figure 2:**
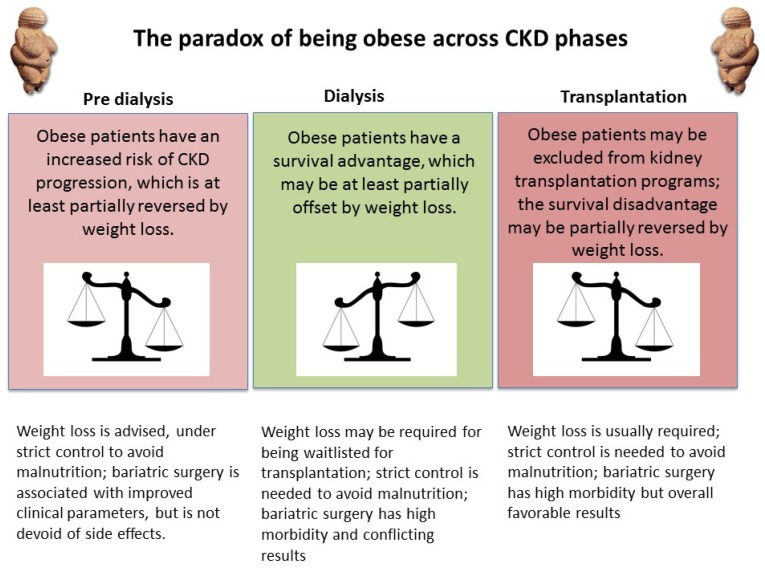
The obesity paradox.

In the overall population obesity is associated with higher morbidity and mortality; hence the longer survival of obese patients in dialysis, the paradigm of reverse epidemiology, was initially surprising [136]. The effect of weight on survival on dialysis produces a u-shaped curve: in the short term, the highest risk is for underweight, malnourished patients, but obese patients, who are initially not affected by malnutrition, may pay a price in terms of mortality and comorbidity in the long run [137]. The association is observed in all dialysis populations, in chronic kidney disease and is modulated by dialysis efficiency, diabetes and sex [136,137,138,139,140] (Figure 2).

While life expectancy is longer after kidney transplantation, high BMI is an important contraindication for transplantation (although not an automatic disqualification in all centers), due to the poorer results obtained in obese patients [141,142,143,144,145,146,147,148]. Thus the paradoxical exclusion of obese patients, who are likely to have a longer life expectancy, from many transplant programs, underlines the many controversial facets of nutrition in dialysis [147].

The balance between the advantages and disadvantages of obesity varies across CKD phases (Figure 2). Obesity increases the risk of progression of CKD, and weight loss is advised in pre-dialysis patients. Obesity displays a survival advantage in dialysis, but represents a barrier for kidney transplantation; the indications to lose weight on dialysis are controversial, leading some authors to suggest that weight loss programs be limited to patients potentially waitlisted for kidney transplantation [138]. It is not easy for dialysis patients to lose weight and the associated benefit is not clear-cut, and may be absent [149,150,151,152]. As the risks of bariatric surgery are increasingly being recognized, the best modality for weight reduction in waitlisted dialysis patients is a matter of controversy [152].

The conflicting pattern of obesity-related risks across CKD phases suggests that obesity is not healthy per se, but it protects the patient from the dangers of malnutrition on dialysis in the same way as it protected our ancestors from dying in times of famine in prehistoric times [153]. In this sense, dialysis may be another example of the advantage of “thrifty” genotypes (Figure 2).

The differences between patterns of overweight/obesity suggest a tailored approach to obese dialysis patients, optimally within a flexible policy of transplant waitlisting [154,155]. Intensifying dialysis, thereby allowing for safer weight loss, is a support policy that should be considered [156].

### 4.2. Second Paradox: Phosphate, Acidosis and Protein Intake

The traditional approach involves starting a high-protein diet (1.2–1.4 g/kg/day of protein) immediately before renal replacement therapy, to compensate for strict pre-dialysis diets. In fact protein loss via haemodialysis, is a problem that has been known since the early years of renal replacement therapy [37,38,39,40,96,97,98,99,157].

This recommendation should be contextualized to the state of our knowledge in the late 70s and early 80s, in a younger dialysis population with late-start dialysis, lower efficiency, pre-erythropoietin, before the catabolic effect of acidosis was fully acknowledged. This may not fully apply to our ageing dialysis population [158,159].

Unfortunately high protein intake is not without side effects: fixed acid production increases on a protein rich diet (in particular one with a preponderance of food of animal origin), as does phosphate load, thus leading to a conflict between ensuring high protein intake or correcting acidosis and hyperphosphatemia [40,41,42,43,44,45,46,47].

Three points need to be underlined in this regard:

- Conflict between protein and phosphate is limited to thrice-weekly dialysis, while more frequent sessions, in particular nightly haemodialysis schedules, can remove so much phosphate that it becomes necessary to add phosphate to the dialysate [48,49,90,91,119,120,121,122,123].
- Not all phosphate is created equal: plant organic phosphate is less absorbable than phosphate in animal protein. Added inorganic phosphate, found in food preservatives, is devoid of nutritional benefit and is more easily absorbed; information on added phosphate is however often lacking from packaging [102,103,104,105,106].
- The role of animal-derived proteins in human nutrition has been reassessed in recent years, yet this critical analysis has not been fully extended to CKD and dialysis patients, for whom the equation “high quality protein = animal-derived protein = best protein” is still the basis for prescription, even if the interest on plant-based diets in all CKD stages is increasing [106,107,108,109,110,111,160,161,162].

**Figure 3:**
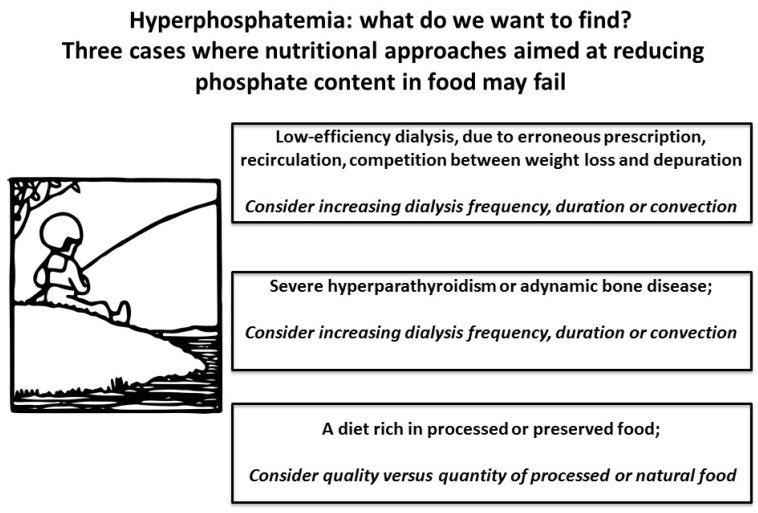
The phosphate, albumin, malnutrition paradox.

Current approaches are more flexible regarding phosphate restriction, and are mainly aimed at avoiding added phosphate, increasingly recognized as unhealthy in CKD patients as well as in the overall population. A more liberal intake of plant-derived phosphate, together with attention to avoiding food additives, is probably compatible with good metabolic control and less need for phosphate binders, which account for a relevant portion of the pill burden in dialysis patients [160,161,162,163,164,165,166,167]. The awareness that hyperphoshatemia reflects a variety of nutritional and non-nutritional derangements can guide tailored interventions [167] (Figure 3).

### 4.3. Third Paradox: High-Efficiency Dialysis and Serum Albumin Levels

For over three decades, insufficient dialysis has been associated with poor outcomes and protein energy wasting [65,66,67,68,69,70,168,169].

High-flux dialysis/haemodiafiltration, with biocompatible membranes and ultrapure water have a higher efficiency profile and are generally better tolerated, as is peritoneal dialysis [170,171,172,173,174,175,176,177,178]. However, one of the prices to pay is the increased loss of amino acids and of small size proteins, that can account for several grams of albumin loss per session [179,180,181].

**Figure 4:**
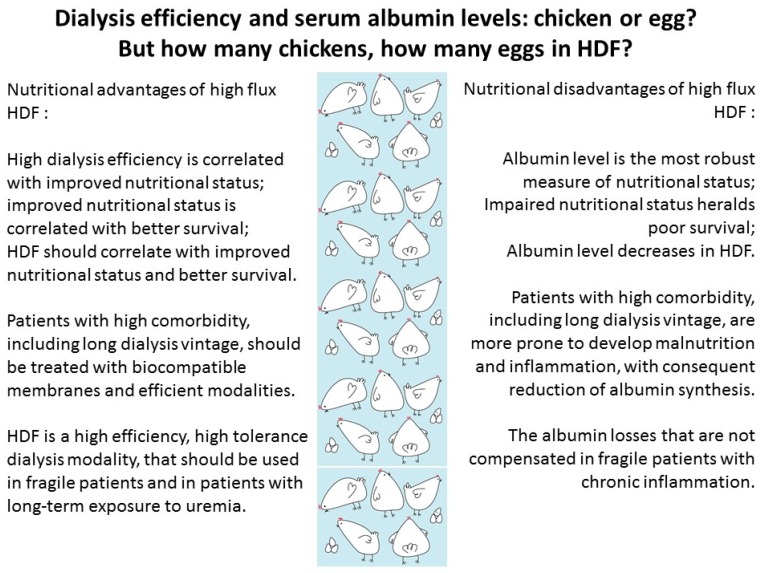
The efficiency-low albumin paradox.

This accounts for another paradoxical situation: the most biocompatible membranes, including the peritoneal one, and the highest ultrafiltration efficiency on extracorporeal dialysis are associated with lower serum albumin levels, hence with a robust marker of poor survival (Figure 4) [179,180,181].

Albumin losses have been quantified differently, and the heterogeneity depends on several factors:

- On extracorporeal dialysis, albumin loss depends on the type of dialysis membranes used, dialysis, hematocrit, albumin and total protein level, blood and dialysate flows; due in part to the difficulty of sampling, few studies have assessed the intersession variability of albumin losses in individual patients, in stable conditions and in specific situations, such as inflammatory states;
- No validated standardized method is available in clinical practice for dosing albumin levels at the low concentrations usually attained in the dialysate;

As will be further discussed, a missing piece of the puzzle is albumin synthesis, which is reduced in patients with chronic inflammation or severe vascular disease, once more linking nutrition and dialysis efficiency with inflammation and atherosclerosis; as some authors claim that prealbumin may be a better nutritional marker in these cases, the controversy is still open [182,183,184].

### 4.4. Fourth Paradox: Potassium and Vascular Health

**Figure 5:**
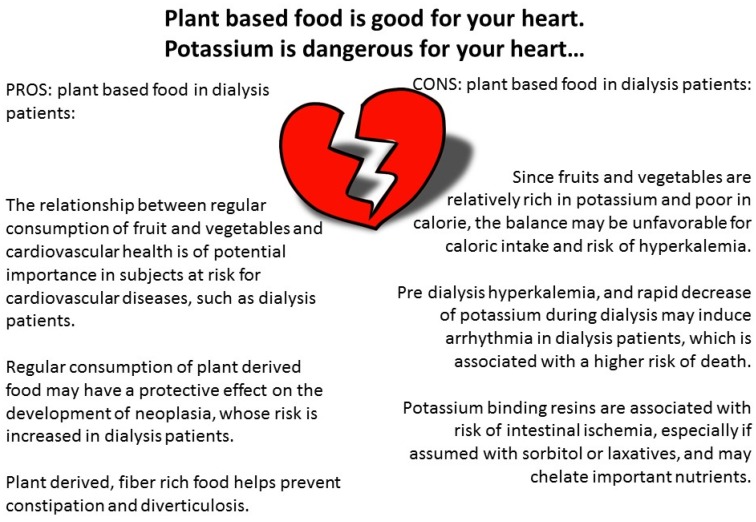
The healthy heart low-potassium paradox.

Seeking healthy nutrition has become fashionable: in most European countries, television and advertisements remind the public that healthy nutrition includes at least two servings of fruit and vegetables per day. The attention paid to the Mediterranean Diet, DASH and vegan-vegetarian diets underlines the importance of an adequate potassium intake for the prevention of cardiovascular diseases and highlights the risks linked to diets overly rich in animal proteins [185,186,187,188,189,190,191,192]. Similar data are observed in CKD patients, in which a “healthy” diet is associated with longer survival [193,194,195]. Yet it is difficult to reconcile the first killer of dialysis patients with the emerging nutritional indications. Although patients on dialysis are generally instructed to avoid fruit and vegetables, potassium is ubiquitous, and protein-rich food is often also rich in potassium. In fact, as was pointed out, limitations on potassium-rich food are not fully supported by the current literature, and a more liberal consumption of plant-derived food is probably to be advised, also because it reduces constipation (Figure 5) [14,22,167]. Once more, attention should be paid to the potassium contained in food preservatives rather than in fresh fruit and vegetables, while careful management of acidosis and tailored dialysis schedules can offer alternatives to dietary restrictions [14,22,23,24,25,26].

The use of potassium binders, potentially useful in liberalizing the “renal diet”, is still matter of discussion, both with the older polystyrene resins, whose use combined with laxatives has been associated with intestinal ischemia, and with the new potassium binders, Patiromer, and Zirconium Cyclosilicate (ZS-9), the first however associated with electrolyte depletion (in particular hypomagnesemia) and the second with an increased risk of edema [196,197,198,199].

## 5. What This Review Did Not Address

This review was undertaken to support the dietitian and nursing team in our hospital (Centre Hospitalier Le Mans, France) by providing a summary of the open problems and ongoing discussions in the clinical management of diet in haemodialysis patients. It does not focused on the main strategies for correcting malnutrition, briefly summarized in Table 3. It did not address many important points, including the diagnosis of malnutrition or protein energy wasting; the organization of the dietitian’s support to the dialysis population; the psychological aspects of malnutrition and its relationship with depression; the role of uremic toxins; the reversibility of malnutrition after kidney transplantation and the role of macrobiotics. The acknowledgement of these limitations underlines the complexity of the issue and the need for continuous updating in this complex and evolving field.

## 6. Conclusions and Suggestions for Future Research

Dialysis and diet are a dyad that cannot be divided if we wish to ensure the best possible metabolic balance in our patients. In taking into account the improvements in dialysis treatment and the demographic changes in the dialysis population, current dietary approaches are less restrictive than those adopted in the past, and flexible and tailor-made diet-dialysis policies are gaining interest.

The vast literature on ways that malnutrition can be prevented or reversed has often produced inconsistent results, indirectly supporting personalized interventions, and demonstrating the need for dedicated, committed and specialized multidisciplinary teams of professionals with nutritional, educational and rehabilitation skills in the core clinical management of dialysis patients.

## Figures and Tables

**Table 1:** A simplified historical scheme of the main focuses in diets for patients on chronic haemodialysis.

Period *	The Main “Enemy”	The Risks	Dietary Indications	Notes
First era	Potassium	Hyperkalemia can be deadly	Restrictions on fruit and vegetables	Can be mitigated by high dialysis efficiency and acidosis control
Second era	Phosphate	Vascular calcification, vascular ageing	Restrictions on cheese, milk and derivates	May contrast with the indications of high-protein intake
Third era	Malnutrition	Risk of death is higher in malnourished patients	Increased protein and calorie intake	May be impossible to conciliate with previous restrictions

* The definition of each period is approximate, as each one merges with the next, and the first warnings on malnutrition are as old as dialysis itself, while we should always keep in mind the short-term risks of hyperkalemia and the long-term importance of hyperphosphatemia.

**Table 2:** A synthesis of the present panorama on diet and dialysis through four open questions and four paradoxes.

The Questions	Pros	Cons	Comments
Are the nutritional requirements usually cited (calories: 30–35 kcal/kg; proteins > 1.2 g/kg) still relevant?	International standard followed for more than 40 years	The requirements were assessed in a different dialysis population, and may not be relevant to the present one; they do not consider the changes in the indications given to the overall population	There is a need for a re-assessment of the requirements of elderly dialysis patients
Are the present standards of “adequate nutrition” applicable to intensive dialysis schedules, and to haemodiafiltration?	Simple markers such as albumin level make it possible to compare results, and are robust enough to maintain a constant predictive value	Sensibility may be lower in non conventional dialysis techniques, and can be affected by albumin losses in haemodiafiltration	None of the proposed evaluations of malnutrition is clearly superior or self-standing; results of studies depend in part on the definition-diagnoses chosen
Processed and preserved food may be significantly different from untreated food. What are we eating?	Nutritional approaches have to be simple and basing them on quantity and quality may not be feasible	Processed foods may be rich in rapidly absorbable phosphate and potassium	Not acknowledging the importance of additives in processed and preserved foods can lead to unnecessary restrictions
Is malnutrition a single disease or the result of several diseases?	The clinical signs of malnutrition are universal and do not depend on pathogenesis	If malnutrition is not linked to poor intake but to poor clinical conditions, itwill not respond to therapy	Differentiation may allow setting attainable goals according to the individualpatient’s comorbidity
The paradoxes	The “logic” (overall population or general data in the dialysis population)	The finding (in the dialysis population or in specific dialysis populations)	Comments
Obesity and survival	Obesity is associated with lower survival in the overall population	Obesity is associated with higher survival in dialysis patients; losing weight is associated with higher mortality on dialysis	Obesity is often a contraindication for kidney transplantation
High protein intake and phosphate control	A high protein diet is indicated after dialysis start	Reduction of phosphate intake is not compatible with a high-protein diet	Plant derived phosphate may be less well absorbed; acidosis induced by catabolism is often a missing element in hyperphosphatemia
Albumin level, Kt/V and survival	Low serum albumin and low dialysis efficiency are associated with reduced survival	In haemodiafiltration, high efficiency is coupled with significant albumin losses	Albumin losses are incompletely quantified; nutrition is probably more important than high efficiency in elderly or fragile sarcopenic patients
Potassium and vascular health	Since dialysis patients are at risk for hyperkalemia, potassium is often restricted	Banning plant derived food to avoid hyperkalemia limits consumption of “vascular healthy” food in a high-risk population	Hyperkalemia is still a rare, but possible cause of death

**Table 3:** A schematic revision of limits and advantages of interventions to improve the nutritional status of dialysis patients.

The Field of Intervention	Intervention	Pros	Cons
*Dialysis optimization*	Increasing efficiency and tolerance by increasing frequency (daily or more frequent dialysis) [200,201,202,203,204,205,206,207,208,209,210,211,212,213]	Improvement in nutritional status in most of prospective studies (see also pregnancy on dialysis)	May be difficult to organize; possibly higher risk of vascular access problems
	Increasing efficiency and probably also tolerance by switching to convective dialysis modalities (such as high flow haemodiafiltration) [214,215,216,217,218,219,220,221,222,223,224,225,226]	Efficiency is associated with nutritional status at least in “standard patients”	Losses may be significant in elderly, malnourished patients. No demonstration of nutritional advantages
	Decreasing losses, and preserving renal function (incremental dialysis, tailored dialysis) [227,228,229,230,231,232,233,234,235,236,237,238,239,240,241,242]	Residual diuresis and residual renal function are two of the most powerful predictors of survival; “dialysis shock” may be a cause of early death after dialysis start	Experience is still limited and there is still no agreed standard
*Physical exercise*	Physical exercise is theoretically a powerful means of improving clinical conditions and nutritional status in patients with a chronic disease [243,244,245,246,247,248,249,250,251,252]	The best results have been reported in observational studies; biases linked to self-selection limit the generalization of results.	Barriers are evident in the elderly population, in which inactivity is often the result of the same comprehensive physical failure that causes malnutrition
*Metabolic interventions* *	Anemia correction [253,254]	ESA improved quality of life, fertility and sex life, issues associated with nutritional status	The association between lack of response to ESAs, inflammation, malnutrition and atherosclerosis is part of the MIA syndrome
	Thyroid hormones [255]	The euthyroid sick syndrome or “low T3 syndrome” is typical of malnutrition/starvation	Correction of the metabolic deficit can worsen the clinical picture
	Androgen steroids [256,257,258,259]	Recently reconsidered therapeutic options include nandrolone decanoate and oxymetholone, which display good effects on sarcopenia	Side effects may be relevant; this treatment could be considered in males with testicular failure and severe sarcopenia
	Recombinant growth hormone [260,261,262,263,264,265,266,267]	Recombinant growth hormone is routinely used in children on dialysis. In adults, growth hormone is often low, and the effect on severe malnutrition has been favorable	High costs and side effects limit its use
*Nutritional interventions*	Increasing the quantity/quality of food [268,269,270,271,272,273,274,275,276,277,278]	The best tool for improving nutritional status, eating during dialysis may be an important way to improve the nutritional status of dialysis patients	If malnutrition is linked to inflammation and atherosclerosis, it is difficult to increase the quantity or quality of food
	Nutritional supplements (oral) [279,280,281,282,283,284,285,286,287,288,289,290,291,292]	Can be of use especially for limited periods of time; specific supplements for dialysis patients (poor in phosphate) are also available	Can decrease appetite for “normal” food; may be less tasty after a longer period
	Intravenous or enteral supplements [293,294,295]	Can help reverse acute malnutrition, especially in the case of failure of the two previous interventions	May further reduce food intake; and create a need for a high quantity of fluids; metabolic derangements are frequent in the long term

* All major metabolic derangements, including acidosis, hyperparathyroidism and hypovitaminosis D, are associated with poor nutritional status and higher mortality in dialysis.
